# Supplementary material for: Salicylic Acid and Risk of Colorectal Cancer: A Two-Sample Mendelian Randomization Study
Source: Nutrients. 2021 Nov 21;13(11):4164. doi: 10.3390/nu13114164 (PMC8620763; doi:10.3390/nu13114164)
Supplement: Supplementary file 1 [file nutrients-13-04164-s001.zip › nutrients-1440990-supplementary.pdf]

Supplementary information

Supplementary Table S1- Enzyme genomic regions based on NCBI Build 37/UCSC hg19.

Gene	Chromosome	Start site	End site
BChE	3	165,490,692	165,555,260
PAFAH1B2	11	117,014,983	117,047,610
PAFAH1B3	19	42,297,033	42,303,546
UGT1A6	2	234,600,253	234,681,946
CYP2C9	10	96,698,415	96,749,147
ACSM2B	16	20,547,548	20,587,749

Supplementary Table S2- Associations of the 4 functional SNPs with salicylic acid

SNP	Metabolite	Effect allele	Other allele	Beta	SE	LCI	UCI	EAF	Chr	Position	P value	Sample size	Gene
**rs6445035**	Salicylic acid	A	G	8.71×10^{-3}	1.49×10^{-2}	-0.02	0.04	0.20	3	165480100	0.56	14149	*BChE*
**rs2070959**	Salicylic acid	G	A	0.01	1.28×10^{-2}	-0.01	0.04	0.32	2	234602191	0.30	14149	*UGT1A6*
**rs1105879**	Salicylic acid	C	A	0.02	1.26×10^{-2}	-6.79×10^{-3}	0.04	0.34	2	234602202	0.16	14149	*UGT1A6*
**rs1799853**	Salicylic acid	T	C	-0.02	1.75×10^{-2}	-0.06	0.01	0.14	10	96702047	0.21	14149	*CYP2C9*

Supplementary Table S3-Pathway SNP associations with salicylic acid

SNP	Effect allele	Other allele	Beta	SE	LCI	UCI	EAF	Direction	Chr	Position	P value	Sample size	LD clumping R^2
**rs8062555**	C	G	-0.27	0.04	-0.35	1.74	0.9792	--	16	20566287	$2.07x10^{-10}$	14149	0.001 and 0.8
**rs9922093**	T	C	-0.18	0.03	-0.24	1.82	0.9666	--	16	20568843	$1.01\ x10^{-07}$	14149	0.001 and 0.8
**rs146980165**	A	T	0.24	0.05	0.15	2.25	0.0168	++	16	20576746	$2.42\ x10^{-07}$	14149	0.8
**rs7499557**	T	G	-0.25	0.04	-0.34	1.75	0.9788	--	16	20557620	$1.29\ x10^{-09}$	14149	0.8
**rs7500194**	A	G	0.08	0.02	0.05	2.06	0.8573	++	16	20549505	$1.90x10^{-06}$	14149	0.8
**rs8056693**	T	C	0.18	0.04	0.11	2.18	0.0298	++	16	20570661	$2.94x10^{-07}$	14149	0.8

Abbreviations: SE, standard error; LCI, lower confidence interval; UCI, upper confidence interval; Chr, chromosome; LD, linkage disequilibrium.

Supplementary Table S4-Pathway SNP associations with colorectal cancer

Sample	SNP	Effect allele	Other allele	Beta	SE	LCI	UCI	EAF	Direction	Chr	Position	P value	Sample size
**GECCO**	rs8062555	C	G	0.012	0.028	-0.043	0.067	0.967	-+-++-	16	20566287	0.67	120328
	rs9922093	T	C	-0.030	0.025	-0.079	0.018	0.967	+-+---	16	20568843	0.22	120328
	rs146980165	A	T	-0.018	0.033	-0.082	0.046	0.026	+-+--+	16	20576746	0.59	120328
	rs7499557	T	G	0.017	0.030	-0.042	0.075	0.969	-+-++-	16	20557620	0.57	120328
	rs7500194	A	G	-0.005	0.013	-0.030	0.020	0.848	+-----	16	20549505	0.71	120328
	rs8056693	T	C	0.035	0.026	-0.016	0.086	0.030	-+-+++	16	20570661	0.18	120328
**DACHS all**	rs8062555	G	C	-0.016	0.110	-0.233	0.200	0.021	NA	16	20566287	0.88	7851
	rs9922093	C	T	0.059	0.092	-0.122	0.240	0.032	NA	16	20568843	0.52	7851
	rs146980165	A	T	0.053	0.125	-0.192	0.299	0.017	NA	16	20576746	0.67	7851
	rs7499557	G	T	0.018	0.113	-0.203	0.238	0.021	NA	16	20557620	0.87	7851
	rs7500194	G	A	0.010	0.046	-0.080	0.099	0.141	NA	16	20549505	0.83	7851

	rs8056693	T	C	0.077	0.096	-0.112	0.266	0.029	NA	16	20570661	0.42	7851
**DACHS aspirin users**	rs8062555	G	C	-0.041	0.244	-0.519	0.438	0.021	NA	16	20566287	0.87	1589
	rs9922093	C	T	0.009	0.196	-0.375	0.393	0.034	NA	16	20568843	0.96	1589
	rs146980165	A	T	-0.044	0.263	-0.559	0.472	0.018	NA	16	20576746	0.87	1589
	rs7499557	G	T	0.056	0.250	-0.433	0.546	0.020	NA	16	20557620	0.82	1589
	rs7500194	G	A	-0.007	0.104	-0.210	0.197	0.131	NA	16	20549505	0.95	1589
	rs8056693	T	C	0.029	0.200	-0.364	0.422	0.033	NA	16	20570661	0.88	1589
**DACHS aspirin non-users**	rs8062555	G	C	-0.025	0.131	-0.282	0.232	0.022	NA	16	20566287	0.85	5,660
	rs9922093	C	T	0.128	0.112	-0.092	0.347	0.031	NA	16	20568843	0.25	5,660
	rs146980165	A	T	0.070	0.152	-0.228	0.368	0.016	NA	16	20576746	0.64	5,660
	rs7499557	G	T	-0.003	0.134	-0.266	0.259	0.021	NA	16	20557620	0.98	5,660
	rs7500194	G	A	0.002	0.054	-0.104	0.109	0.142	NA	16	20549505	0.96	5,660
	rs8056693	T	C	0.147	0.118	-0.084	0.378	0.028	NA	16	20570661	0.21	5,660

Supplementary Table S5- Pathway SNP associations with CRC using the other MR methods

Study	Method	N SNPs	OR	LCI	UCI	P value
**GECCO**	MR Egger	6	0.99	0.78	1.27	0.97
	MR Egger (accounting for LD correlation)		1.15	0.98	1.36	0.09
	Weighted median		0.95	0.83	1.09	0.45
	Inverse variance weighted		1.01	0.91	1.12	0.88
	Weighted mode		0.94	0.80	1.12	0.53
**DACHS all**	MR Egger	6	1.25	0.52	3.02	0.64
	MR Egger (accounting for LD correlation)		0.27	0.11	0.67	4.78×10^{-3}
	Weighted median		1.11	0.70	1.75	0.67
	Inverse variance weighted		1.14	0.77	1.68	0.53
	Weighted mode		1.01	0.55	1.84	0.98
**DACHS aspirin users**	MR Egger	6	0.95	0.13	6.69	0.96
	MR Egger (accounting for LD correlation)		0.02	2.17×10^{-3}	0.13	9.45×10^{-5}
	Weighted median		1.06	0.39	2.91	0.90
	Inverse variance weighted		1.02	0.44	2.40	0.96
	Weighted mode		1.14	0.32	4.03	0.84
**DACHS aspirin non-users**	MR Egger	6	1.26	0.44	3.61	0.68
	MR Egger (accounting for LD correlation)		0.46	0.18	1.13	0.09
	Weighted median		1.02	0.57	1.83	0.94
	Inverse variance weighted		1.26	0.78	2.01	0.35
	Weighted mode		0.97	0.45	2.11	0.94

Abbreviations: OR, odds ratio; LCI, lower confidence interval; UCI, upper confidence interval.

Supplementary Table S6- Results of the Q statistic heterogeneity test for pathway SNPs

Study	Method	Q statistic	Degrees of freedom	P value
**GECCO**	MR Egger	4.20	4	0.38
	IVW	4.21	5	0.52
**DACHS all**	MR Egger	0.86	4	0.93
	IVW	0.92	5	0.97
**DACHS aspirin user**	MR Egger	0.12	4	1.00
	IVW	0.13	5	1.00
**DACHS aspirin non-user**	MR Egger	2.21	4	0.70
	IVW	2.21	5	0.82

Supplementary Table S7- MR Steiger test results

Analysis	Dataset	Clumping (R2)	N SNPs	snp_r2.exposure	snp_r2.outcome	correct_causal_direction	steiger_pval
**Pathway**	GECCO	0.001	2	0.1019819	0.00106105	TRUE	5.50E-247
		0.8	6	0.263845	0.00291445	TRUE	0
	DACHS all	0.001	2	0.1019819	0.003752854	TRUE	9.16E-82
		0.8	6	0.263845	0.01296463	TRUE	1.38E-227
	DACHS aspirin user	0.001	2	0.1019819	0.001731796	TRUE	8.82E-28
		0.8	6	0.263845	0.007709451	TRUE	2.49E-73
	DACHS aspirin non-user	0.001	2	0.1019819	0.01694397	TRUE	4.93E-37
		0.8	6	0.263845	0.04351382	TRUE	2.23E-113
**Genome-wide**	GECCO	0.001	1	0.0170075	9.41E-05	TRUE	1.64E-42
		0.8	4	0.1846267	0.00158571	TRUE	0.00E+00
	DACHS all	0.001	1	0.0170075	2.97E-06	TRUE	3.71E-20
		0.8	4	0.1846267	0.002836863	TRUE	3.93E-183
	DACHS aspirin user	0.001	1	0.0170075	0.002832002	TRUE	0.003265884
		0.8	4	0.1846267	0.01445678	TRUE	1.86E-37
	DACHS aspirin non-user	0.001	1	0.0170075	0.000210312	TRUE	1.21E-13
		0.8	4	0.1846267	0.008855774	TRUE	3.57E-119

Supplementary Table S8- Genome-wide SNP associations with salicylic acid

SNP	Effect allele	Other allele	Beta	SE	LCI	UCI	EAF	Direction	Chr	Position	P value	Sample size	LD clumping R^2
**rs7498776**	T	C	-0.13	0.02	-0.17	-0.09	0.90	--	16	20611149	8.70 x10^{-11}	14149	0.001 and 0.8
**rs11642648**	A	G	0.18	0.03	0.11	0.24	0.04	++	16	20610955	3.58x10^{-08}	14149	0.8
**rs7499557**	T	G	-0.25	0.04	-0.34	-0.17	0.98	--	16	20557620	1.29x10^{-09}	14149	0.8
**rs8062555**	C	G	-0.27	0.04	-0.35	-0.18	0.98	--	16	20566287	2.07 x10^{-10}	14149	0.8

Abbreviations: SE, standard error; LCI, lower confidence interval; UCI, upper confidence interval; Chr, chromosome; LD, linkage disequilibrium.

Supplementary Table S9- Genome-wide SNP associations with colorectal cancer

Sample	SNP	Effect allele	Other allele	Beta	SE	LCI	UCI	EAF	Direction	Chr	Position	P value	Sample size
**GECCO**	rs7498776	T	C	-0.010	0.015	-0.039	0.019	0.895	--++--	16	20611149	0.5122	120328
	rs11642648	A	G	0.033	0.024	-0.014	0.079	0.036	-+-+++	16	20610955	0.1678	120328
	rs7499557	T	G	0.017	0.030	-0.042	0.075	0.969	-+-++-	16	20557620	0.5746	120328
	rs8062555	C	G	0.012	0.028	-0.043	0.067	0.967	-+-++-	16	20566287	0.6747	120328
**DACHS all**	rs7498776	C	T	0.002	0.055	-0.106	0.109	0.093	NA	16	20611149	0.974898	7851
	rs11642648	A	G	0.047	0.089	-0.127	0.222	0.034	NA	16	20610955	0.595083	7851
	rs7499557	G	T	0.019	0.113	-0.202	0.239	0.021	NA	16	20557620	0.867246	7851
	rs8062555	G	C	-0.015	0.110	-0.232	0.201	0.021	NA	16	20566287	0.890538	7851
**DACHS aspirin users**	rs7498776	C	T	-0.053	0.121	-0.291	0.185	0.093	NA	16	20611149	0.660838	1589
	rs11642648	A	G	0.082	0.193	-0.296	0.461	0.035	NA	16	20610955	0.66917	1589
	rs7499557	G	T	0.056	0.250	-0.433	0.546	0.020	NA	16	20557620	0.821499	1589
	rs8062555	G	C	-0.041	0.244	-0.519	0.438	0.021	NA	16	20566287	0.868093	1589
**DACHS aspirin non-users**	rs7498776	C	T	0.015	0.065	-0.113	0.142	0.094	NA	16	20611149	0.823269	5,660
	rs11642648	A	G	0.090	0.108	-0.121	0.300	0.033	NA	16	20610955	0.404607	5,660
	rs7499557	G	T	-0.003	0.134	-0.265	0.259	0.021	NA	16	20557620	0.982292	5,660
	rs8062555	G	C	-0.025	0.131	-0.282	0.233	0.022	NA	16	20566287	0.851039	5,660

Supplementary Table S10- Genome-wide SNP associations with CRC using the other MR methods

Study	Method	N SNP	OR	LCI	UCI	P value
**GECCO**	MR Egger	4	0.84	0.58	1.22	0.46
	MR Egger (accounting for LD correlation)	4	1.16	0.99	1.38	0.08
	Weighted median	4	1.00	0.87	1.14	0.96
	Inverse variance weighted	4	1.03	0.92	1.15	0.67
	Weighted mode	4	0.96	0.80	1.16	0.73
**DACHS all**	MR Egger	4	1.00	0.24	4.24	1.00
	MR Egger (accounting for LD correlation)	4	0.11	0.04	0.28	4.52×10^{-6}
	Weighted median	4	1.03	0.64	1.65	0.90
	Inverse variance weighted	4	1.06	0.69	1.63	0.79
	Weighted mode	4	1.01	0.54	1.89	0.98
**DACHS aspirin users**	MR Egger	4	1.60	0.07	38.99	0.80
	MR Egger (accounting for LD correlation)	4	2.47×10^{-3}	2.37×10^{-4}	0.03	5.07×10^{-7}
	Weighted median	4	0.97	0.32	2.96	0.96
	Inverse variance weighted	4	0.99	0.38	2.57	0.99
	Weighted mode	4	0.85	0.22	3.29	0.83
**DACHS aspirin non-users**	MR Egger	4	0.82	0.15	4.55	0.84
	MR Egger (accounting for LD correlation)	4	0.23	0.10	0.52	4.15×10^{-4}
	Weighted median	4	1.03	0.58	1.83	0.92
	Inverse variance weighted	4	1.10	0.66	1.84	0.71
	Weighted mode	4	0.99	0.47	2.06	0.98

Abbreviations: OR, odds ratio; LCI, lower confidence interval; UCI, upper confidence interval.

Supplementary Table S11- Results of the Q statistic heterogeneity test for genome-wide SNPs

Study	Method	Q statistic	Degrees of freedom	P value
**GECCO**	MR Egger	1.46	2	0.48
	IVW	2.64	3	0.45
**DACHS all**	MR Egger	0.26	2	0.88
	IVW	0.26	3	0.97
**DACHS aspirin users**	MR Egger	0.36	2	0.84
	IVW	0.45	3	0.93
**DACHS aspirin non-users**	MR Egger	0.52	2	0.77
	IVW	0.64	3	0.89
